# Retromer stabilization using a pharmacological chaperone protects in an α-synuclein based mouse model of Parkinson’s

**DOI:** 10.21203/rs.3.rs-3417076/v1

**Published:** 2023-10-19

**Authors:** Simona Eleuteri, Tracy Shi Zhang Fang, Gianni Cutillo, Michele Persico, David K Simon

**Affiliations:** Beth Israel Deaconess Medical Center; Beth Israel Deaconess Medical Center; Beth Israel Deaconess Medical Center; Beth Israel Deaconess Medical Center; Beth Israel Deaconess Medical Center

**Keywords:** αSynuclein pathology, Parkinson’s disease, Pharmacological approach, Retromer stabilization, VPS35

## Abstract

**Background:**

In the present study we assessed the protective effects of a pharmacological approach to stabilize the retromer complex in a PD mouse model. Missense mutations in the VPS35 gene are a rare cause of familial PD. The VPS35 protein is a subunit of the retromer cargo recognition complex and has a variety of functions within neurons, many of which are potentially relevant for the pathophysiology of PD. Prior studies have revealed a role for the retromer complex in controlling accumulation and clearance of α-synuclein aggregates. We previously identified an aminoguanidine hydrazone, 1,3 *phenyl bis guanylhydrazone* (compound 2a), as a pharmacological stabilizer of the retromer complex that increases retromer subunit protein levels and function.

**Methods:**

Here, we validate the efficacy of 2a in protecting against αSynuclein pathology and dopaminergic neuronal degeneration in a PD mouse model generated by unilateral injection of AAV-A53T-αSynuclein in the substantia nigra.

**Results:**

Daily intraperitoneal administration of 2a at 10 mg/Kg for 100 days led to robust protection against behavioral deficits, dopaminergic neuronal loss and loss of striatal dopaminergic fibers and striatal monoamines. Treatment with 2a activated αSynuclein degradation pathways in the SN and led to significant reductions in aggregates and pathological αSynuclein.

**Conclusion:**

These data suggest retromer stabilization as a promising therapeutic strategy for Parkinson’s disease leading to neuroprotection of dopaminergic neurons and rescue in the accumulation of pathological and aggregates αSynuclein. We identified 2a compound as potential clinical drug candidate for future testing in Parkinson’s disease patients.

## Background

Parkinson’s disease (PD) is the second most common neurodegenerative disorder affecting 1% of people over 65 and 4.3% of those older than 85 [1]. A key pathological feature of PD is loss of dopaminergic (DA) neurons in the substantia nigra pars compacta (SNpc) resulting in degeneration of the nigrostriatal tract and dopamine (DA) deficiency that contributes particularly to the motor features of PD [2]. The neuropathological hallmark of PD is the occurrence of intracellular inclusions, Lewy bodies and Lewy neurites, with a topologically predictable progression [3], which include aggregated αSynuclein (αSyn) [4]. A convincing set of data from genetic, animal models and biochemical studies supports the view that accumulation and aggregation of αSyn in DA neurons represents a key pathogenic event [5].

The VPS35-D620N mutation is a cause of late-onset autosomal dominant PD [6–7]. The VPS35 gene encodes a component of the heteropentameric retromer complex, which mediates retrograde transport of cargo proteins from endosomes to the trans-Golgi network, plasma membranes [8] and mitochondria to peroxisomes [9]. VPS35 is a subunit of the cargo recognition complex, formed by VPS35, VPS26 and VPS29, which has a variety of functions within neurons, many of which are potentially relevant for the pathophysiology of PD [10]. In particular VPS35 controls the regulation and maintenance of neuronal signaling events: i) downregulation of some receptors [11], ii) synaptic plasticity [12–13], iii) trafficking of proteins in dendritic spines [14] and iv) DA transporter (DAT) recycling in DA neurons [15]. VPS35 also is a putative modulator of mitochondrial dynamics, fusion and fission [16–18], and controls the accumulation and clearance of pathological forms of αSyn [19–21].

Several lines of evidence indicate that VPS35 influences αSyn accumulation, including: i) αSyn accumulation in induced pluripotent stem cell (iPSC)-derived tyrosine hydroxylase-positive (TH^+^) DA neurons generated from PD patients with the VPS35-D620N mutation [22]; iii) co-localization of VPS35 and αSyn in intracellular inclusions in PD patient brains [19]; iv) αSyn accumulation upon reduction of VPS35 protein levels in αSyn-overexpressing PD mice [19]; v) accumulation of αSyn and degeneration of DA neurons in the SN of VPS35 deficient or VPS35-D620N mutant mice [20]. On the other hand, genetic manipulations to increase expression of wild type VPS35 attenuates the accumulation of αSyn aggregates and reduces neuronal loss and astrogliosis in a transgenic PD mouse model overexpressing αSyn [19].

The molecular mechanisms leading to neuroprotection by VPS35 against αSyn accumulation are uncertain, but recent studies highlight a pivotal role for VPS35 in control of the main αSyn clearance pathways [10].

Recently retromer dysfunction has been linked to different neurodegenerative disorders, with VPS35 protein levels found to be reduced in vulnerable regions in Alzheimer’s disease (AD) [23], Pick disease, progressive supranuclear palsy [24] and Amyotrophic lateral sclerosis (ALS) [25]. Pharmacological stabilization of the retromer complex increases retromer subunit levels in the CNS and protects against neurodegeneration in ALS [25] and AD [26] mouse models. Starting from a previously published compound R55 [27], we identified a pharmacological chaperone, an aminoguanide hydrazone, *1,3 bis phenyl guanylhydrazone (2a)*, as retromer stabilizer that binds the retromer complex at an active binding site between the VPS29 and VPS35 subunits and leads to a nearly 2-fold increase in protein levels of these 2 subunits *in vitro* and *in vivo* in the CNS [25, 27]. In SOD1^G93A^ mice, daily IP administration of 2a leads to: i) increased protein levels of retromer subunits in spinal cord ventral horn MNs; ii) attenuation of locomotor impairment; iii) increased MN survival and protection of sciatic nerves; iv) significant reduction in SOD1 aggregates and in the total ubiquitinated protein profile; v) increased protein levels of two VPS35 well-known cargoes, Sortilin and CI-MPR [28–29], associated with an increase in mature cathepsin D (CTSD) in MNs [25].

In the current study we demonstrate neuroprotective efficacy of 2a in blocking DA neuronal degeneration and nigrostriatal tract and αSyn pathology in a PD mouse model overexpressing A53T-αSyn following unilateral injection into the SN of AAV-A53T-αSyn [30]. Daily IP administration of 2a at 10 mg/Kg protects against behavioral deficits, loss of DA neurons in the SNpc and loss of striatal dopaminergic fibers and striatal monoamines. Treatment with 2a also activates the main αSyn degradation pathways and reduces total and pathological αSyn aggregates.

## Methods

### Materials

AAV1/2-CMV/CBA-Human A53T αSyn-WPRE-BGH-polyA (GD1001-RV) and AAV1/2-CMV/CBA-Empty-WPRE-BGH-polyA (GD1004-RV) vectors were purchased from Vigene Biosciences. The following antibodies were used for immunostaining or immunoblotting; anti-VPS35 (Abcam, ab157220), anti-VPS26 (Abcam, ab23892), anti-phosphorylated (pSER 129), αSyn (Abcam, ab184674), anti- αSyn (BD Biosciences, clone 42, mouse monoclonal),anti-TH (PelFreez Biologicals, P60101), anti-TH (Millipore, catalog # AB152), anti-CI-MPR (NovusBio, 300-514-2G11), anti-p62 (BD Biosciences, 610497), anti-LC3B (Cell Signaling, D11), anti-Hsc70 (Proteintech, 10654-1-AP), anti-LAMP2A (Santacruz, H4B4 sc-18822), anti-CTSD (Abcam, ab75852); anti-Sortilin (Abcam, ab166640, rabbit polyclonal), anti-β-Actin (Millipore, A5441, mouse monoclonal). Alexa Fluor secondary antibodies were from Invitrogen; HRP-conjugated secondary antibodies were from Cell signaling technology; Biotinylated secondary antibody from Vector Laboratories. The BLOXALL, ABC kit, DAB kit and Vectashield anti-fading mounting medium were purchased from Vector Laboratories.

### Animals

All mice were housed under a 12-hour light/12-hour dark cycle with ad libitum supply of standard chow. The ambient temperature was 21 ± 2°C, and the humidity 55 ± 10%. Mice were housed using a stocking density of 3–5 mice per cage in individually ventilated caging. Mouse studies at BIDMC were approved by the local Institutional Animal Care and Use Committee (IACUC).

### Aims and Study Design

**Figure 1:**
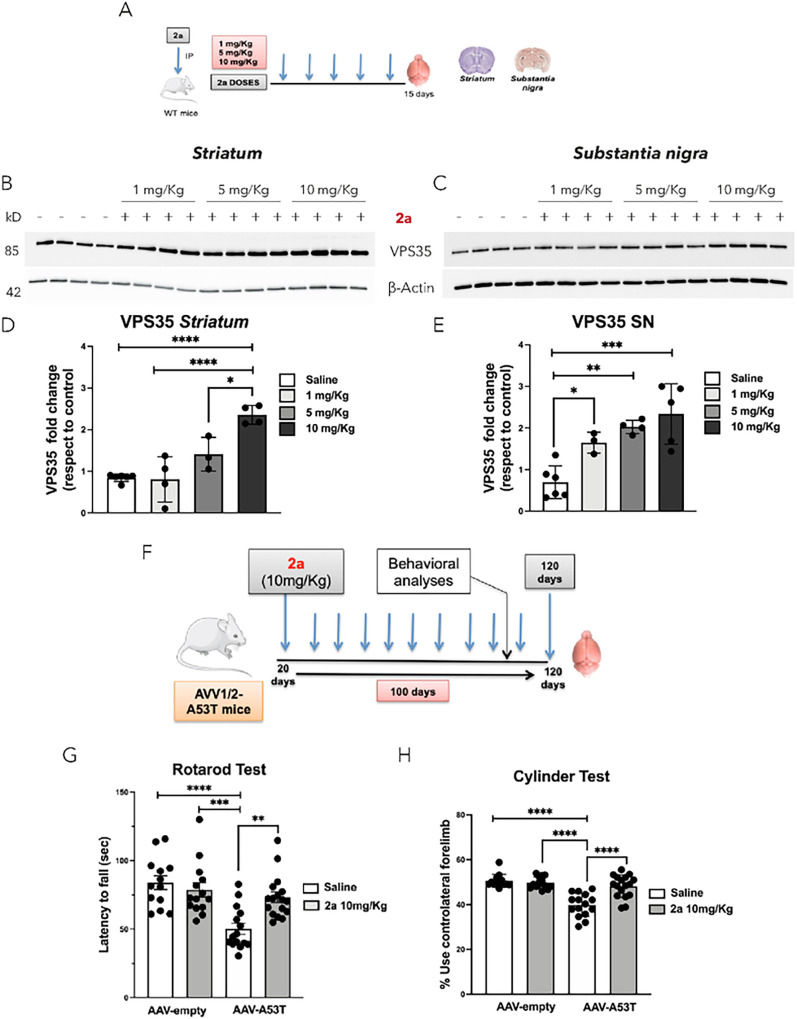
**Assessment of 2a compound in increasing VPS35 protein levels in PD-related areas in wild type mice and on the rescue of behavioral defects in** AAV-A53T-αSyn **mice :** A) Experimental plan of daily IP administration of 2a at three doses (1 mg/Kg, 5 mg/Kg and 10 mg/Kg) for 15 days in wild type mice and protein analysis in the *striatum* and *SN*; B-E) Analysis of VPS35 protein levels upon the administration of 2a at three different doses and normalization with β-actin protein levels in the striatum and SN; significance determined by one-way ANOVA. Error bars represent mean ± SD; F) Experimental plan of the administration of 2a in the AAV-A53T-αSyn PD mouse model. Wild-type mice were unilaterally injected with a control viral vector (AAV-empty) or the A53T vector (AAV-A53T-αSyn) in the SN. We began IP administration of saline or 2a (10 mg/Kg) for 100 days starting at 20 days post-AAV-injection. At 120 days after the AAV injection brain samples were collected and used for biochemical and immunostaining analyses; G) Graph shows statistical analysis of Rotarod test for assessing the efficacy of 2a on motor function of mice at 120 days post-injection of AAV vectors (n= 16 mice per group);H) Graph shows percentage of contralateral forelimb use for rearing in the Cylinder test of mice at 120 days post – injection of AAV vectors (n= 16 mice per group). Significance determined by one-way ANOVA. Error bars represent mean ± SD.

Aims: First compound 2a was assessed for its ability to increase retromer-subunit protein levels in PD-related areas (striatum and SN) as shown in Fig. 1A. Subsequently we tested the impact of pharmacological stabilization of the retromer complex by 2a in an αSyn-based mouse model of PD [30]. To generate the PD mouse model, wild type mice at 12 weeks of age were injected stereotaxically into the SN with AAV1/2 -A53T-αSyn vector or AAV1/2 empty control, and then beginning 20 days after the AAV injection an investigator blinded to the prior procedure started daily IP injections of 2a or saline as a control for 100 days. Behavioral analyses to evaluate motor function were performed by Rotarod and Cylinder tests at 113 to 120 days post stereotaxic AAV injection. At 120 days post injection (after 100 days of daily IP injections with 2a or saline control) neuropathological analyses were performed in striatum and SN. In fixed brains we assessed the effect of 2a on: i) protection against αSyn-induced loss of DA (TH+) neurons in the SN, ii) protection of striatal TH^+^ fibers, iii) retromer subunit immunoreactivity (VPS35 and VPS26) in TH^+^ neurons, iv) retromer function by analyzing immunoreactivity of CI-MPR, a retromer cargo protein, in TH^+^ SN neurons, and v) pathological αSyn (p-Ser-129 αSyn) accumulation. In fresh (unfixed) striatum and SN brain tissues we analyzed the effect of 2a on: i) total and oligomeric αSyn in the SN, ii) striatal monoamines, and iii) αSyn degradation pathways, including CMA, macroautophagy and lysosomal pathways (Fig. 1F).

### Adeno-associated vectors (AAV) 1/2 stereotaxic injection

C57BL/6 mice at 12 weeks of age were anesthetized with ketamine/xylazine and placed in a stereotaxic frame (myNeurolab, Leica Microsystems) with a mouse adapter. 2μl of suspended AAV vector (1 × 10^10^ viral genome copies) was delivered at a speed of 50 nl/second through a pulled glass micropipette pipette (World Precision Instruments) into the right SN with stereotaxic coordinates (AP: −3.0mm, ML: −1.3mm, DV: +4.7mm). AAV1/2-CMV/CBA-Human A53T aSyn-WPRE-BGH-polyA (GD1001-RV) and AAV1/2-CMV/CBA-Null/Empty-WPRE-BGH-polyA (GD1004-RV) vectors were purchased from Vigene Biosciences. After a 5-minute period where the pipette lied in place after dispensation of the virus, the pipette was retracted slowly [30]. Beginning 20 days after the AAV1/2 injection we administrated 2a (or saline control) by daily IP injections (10 mg/Kg) for 100 days in 4 groups of mice: control (empty AAV1/2 vector) vs AAV1/2 -A53T-αSyn vector, each treated with either saline or 2a.

### Behavioral assessment

Cylinder test: Spontaneous forelimb usage was assessed by the cylinder test at 120 days after unilateral AAV injection in 4 groups: AAV1/2-empty and AAV1/2 -A53T-αSyn vectors treated with saline or 2a compound. Mice were placed in a transparent plexiglass cylinder of 12 cm diameter and 30 cm height and were video recorded for 10 minutes or 30 times of rearing, whichever came first. Rearings of the mice were analyzed for the number of touches of the inner surface of the cylinder with either the right (ipsilateral), the left (contralateral) or both forelimbs simultaneously. The final data were presented as the percentage of contralateral forelimb use by calculation with the equation: (contralateral forelimb + both forelimbs)/(contralateral forelimb + ipsilateral forelimb + both forelimbs × 2) × 100. The calculated percentage reflects the degree of asymmetric use of the affected forelimb as follows: 50% = symmetric use of both forelimbs; <50% = preference for the intact (ipsilateral) forelimb; >50% = preference for the affected (contralateral) forelimb. Furthermore, we provided two representative videos recorded during the execution of the cylinder test to evaluate the efficacy of 2a compound in the rescuing the asymmetry in AAV1/2 -A53T-αSyn mice daily treated with 2a compound (10 mg/Kg) compared to the control mice with saline solution. The use of these videos for publication has been approved by the local Institutional Animal Care and Use Committee (IACUC) at BIDMC.

### Rotarod test

Motor activity of mice receiving 2a or vehicle was assessed 113 days after the AAV injection. Briefly, mice were trained for 1 min on a static rotor and for 1 min at constant speed (4 rpm) two times and then two trials were performed over two consecutive days (one per day). Each trial consisted of 3-test sessions with 15 min intervals between sessions. For each session, mice were placed on an accelerating rotor (4–40 rpm) and the latency to fall was recorded, with a maximum limit set at 300 seconds.

### 2a synthesis

The new pharmacological chaperone *1,3 bis phenyl guanylhydrazone* (2a) has been previously designed, synthesized, and patented [25] starting from R55 [27]. Thiophane scaffold has been replaced with a phenyl ring and isothioreas has been substituted with guanylhydrazone to increase the permeability through lipophilic biomembranes [25]. 2a was synthetized by MedKoo Biosciences, the purity of the compound is 99%, structure and molecular weight have been confirmed by ^1^H-NMR and MS analyses. The lyophilized powder stored at −80°C. 2a powder was solubilized in saline solution at 10 mg/Kg prior for the daily IP administration.

### Immunohistochemistry and immunofluorescence staining

For histological studies, the brain was removed after transcardial perfusion with phosphate buffered saline (PBS) followed by 4% paraformaldehyde in PBS (pH 7.0). The brains were then post-fixed overnight in 4% paraformaldehyde in PBS before being immersed in 30% sucrose in PBS to cryopreserve the brains in preparation for cryostat sectioning. Immunostaining was performed on 40-μm thick free-floating coronal sections. For DAB staining, the brain sections were pretreated with BLOXALL (Vector Laboratories) for 10 minutes to exhaust endogenous peroxidases. The brain sections for the double immunofluorescence in DA neurons were performed by incubating with the following primary antibodies: anti-VPS35 (Abcam, ab157220, rabbit monoclonal), anti-VPS26 (Abcam, ab23892, rabbit polyclonal), anti-phosphorylated (pSER 129), αSyn (Abcam, ab184674, mouse monoclonal), anti-TH (PelFreez Biologicals, P60101, sheep polyclonal), anti-CI-MPR (NovusBio, 300–514 (2G11), mouse monoclonal) at 4 °C degrees overnight. We used as secondary antibodies: anti-rabbit-488, anti-mouse-488 and anti-sheep-594.

For the immunohistochemistry the sections were incubated with the primary antibody anti-TH (Millipore, AB152, rabbit polyclonal), and the sections were processed with secondary antibody incubation with or without an ABC kit (Vector Laboratories) and 3’-diaminobenzidine as a chromogen (DAB, Vector Laboratories) on the next day. The sections were mounted on Superfrost Plus Microscope Slides (Fisher Scientific) and coverslipped with Vectashield antifading mounting medium (Vector Laboratories) for fluorescence imaging or dehydrated and coverslipped with Permaslip medium (Alban Scientific) for neuronal counting. For immunofluorescence sections were visualized using confocal microscopy, Leica DM6 CS equipped with ×10, ×20, and ×60 objectives and equipped with super-sensitive HyD detectors. Fluorescence signals were recorded as square 8-bit images (1024 × 1024 pixels) and images were post-processed to generate maximal projections of Z-stacks (acquired with a 0.4–0.8 μm step) and cross-sectional profiles of the Z-stack (acquired with a 0.3 μm step) and pseudo-colored using Leica.

### Neuronal Counting and densitometry

For neuronal counting, the midbrain of each mouse was sectioned using a Leica cryostat machine into six series of 40 μm coronal sections and one series was stained with anti-TH antibodies for DA (TH+) neuronal counting. The serial brain sections were then scanned with NanoZoomer XR Digital slide scanner (Hamamatsu) for neuronal counting with the QuPath v0.2.0 software ((https://qupath.github.io) as previously described [30].

For striatal densitometry, TH-stained sections were imaged using a light microscope fitted with a camera (SPOT). Images were captured using a 10 × objective and fixed exposure settings. Densitometry was performed to quantify the intensity of TH immunostaining in the striatum ipsilateral to the stereotaxic injection and in the contralateral striatum. The average intensity of TH staining in a fixed region of the striatum was quantified with ImageJ software (NIH). The relative optical density of TH + fibers was normalized by subtracting the background intensity of the cortex, and the percentage of ipsilateral over contralateral relative density was calculated and used for statistical analysis. Analysis determines the percentage of positive staining area [%area = (stained area/region area) * 100)]. To calculate % of stained area, the same threshold value was applied to all sections with the same antibody staining. All pixels above the threshold value were considered positive. All striatal densitometry and neuronal counting data for the mice were obtained by an investigator blinded to treatment groups.

### Immunoblot

Brain tissues (*SN* and *striatum*) were dissected from saline perfused mice and rapidly homogenized in 250 μL of RIPA lysis buffer (Millipore 20.188; 50mM Tris-HCl pH 7.4; 150mM NaCl, 0.25 deoxycholic acid, 1% NP40), 0,5% of Triton-X100 and protease inhibitor cocktail (Sigma 100X). Brain extracts were homogenized using a tight-fitting glass Potter tissue grinder (1 ml; Wheaton) and then sonicated at a frequency of 20 kHz (10 times-1s). Brain samples were ultracentrifuged at 120K × g for 30 minutes at 4°C and the supernatants collected as detergent-*soluble fraction*; pellets were resuspended in 300 μL of Ripa buffer, 2% of SDS and Protease Inhibitors, homogenized and sonicated as above and centrifuged at 17K x g for 30 minutes at 4°C and supernatants were collected as the SDS-*soluble fractions*. In the enriched detergent-*soluble* and SDS-*soluble fractions* protein concentration was measured using the BCA protein assay kit. Next, for each sample, 20 μg of protein extract from the SN was loaded on 4–12% Midi-PROTEAN TGX Stain-Free precast gels for PAGE (Bio-Rad). Gel electrophoresis was performed at 200 V for 45 minutes and then protein transferred on nitrocellulose membranes (0,4μm pore). Blots were blocked in 5% of milk in PBS and 0.1% Tween-20 (PBS-T) for 1 hour before exposure to primary antibodies that are incubated overnight at 4°C on a shaker. For the analysis of αSyn aggregates we blotted detergent-soluble and SDS-soluble fractions with anti-αSyn antibody (BD Biosciences, clone 42, mouse monoclonal). For the analysis of αSyn degradation pathways the detergent-soluble fractions were blotted with anti-p62 (BD Biosciences, 610497, mouse purified), anti-LC3B (Cell Signaling, D11, rabbit polyclonal), anti-Hsc70 (Proteintech, 10654-1-AP, rabbit polyclonal), anti-LAMP2A (SantaCruz, H4B4 sc-18822, mouse monoclonal) and anti-CTSD (Abcam, ab75852, rabbit monoclonal); CTSD protein levels were revealed by exposing the membrane for 20 minutes to 0.4% PFA and blocking with 5% of BSA in PBS-T. Retromer complex subunits were detected using anti-VPS35 (Abcam, ab157720 N-terminal, rabbit polyclonal), anti- VPS26 (Abcam, ab23892, rabbit polyclonal) and retromer function studied using anti-Sortilin antibody (Abcam, ab166640, rabbit polyclonal); all antibodies were normalized using anti-β-Actin (Millipore, A5441, mouse monoclonal). Blots were incubated in appropriate HRP-labeled secondary antibodies for 1 hour at room temperature and visualization of protein bands was performed using ClarityMax-ECL (Bio-Rad) according to the manufacturer’s directions and images were acquired on ChemiDoc (Bio-Rad). Quantifications were done using the Image Lab 6.0.1 software (Bio-Rad).

### Measurement of DA and DA metabolites

Mice were sacrificed at 17 weeks post-AAV-A53T or AAV-empty stereotaxic injection in the SN and after 100 days of administration of 2a compound (10mg/Kg) or saline as control. The brains were rapidly removed and placed into a chilled brain matrix and sliced into 1 mm thick coronal sections on ice. The sections were then placed in ice-cold saline. The striatum was dissected from these 1mm sections, snap-frozen and used for HPLC analysis of DA and its metabolites at the Neurochemistry Core, School of Medicine, Vanderbilt University. Levels of DA and its metabolites were normalized to ng/mg of protein input and statistically analyzed with GraphPad Prism software.

### Statistical analysis

We assessed normality of data applying either Kolmogorov–Smirnov test (with Dallas–Wilkinson–Lille for P value) or D’Agostino & Pearson omnibus normality test. For comparisons of the mean differences between 2 groups we used unpaired t-tests. When 3 or more groups were compared, we used analysis of variance (ANOVA) tests followed by Tukey’s multiple Comparison test or by Bonferroni multiple comparison test. Statistical analyses were performed using PRISM 8 (GraphPad Software). P-values less than 0.05 were considered statistically significant.

## Results

### 2a increases VPS35 retromer-subunit protein levels in PD-related brain areas

We previously demonstrated that the retromer stabilizer 2a crosses the blood brain barrier (BBB) and increases VPS35, VPS26 and VPS29 retromer-subunit protein levels in the spinal cord of wild type mice and in an ALS mouse model (SOD1^G93A^) [25]. To assess the effect of 2a in increasing retromer-subunit protein levels in PD-related brain areas (striatum and SN), 2a was administrated by daily IP injections at three different doses (1 mg/Kg, 5 mg/Kg and 10 mg/Kg) for 15 days in wild type mice (Fig. 1A). After 15 days the brains were dissected and analyzed for VPS35 protein levels in the striatum (Fig. 1B & D) and SN by immunoblot (Fig. 1C & E). Administration of 2a at 5 mg/Kg and 10 mg/Kg significantly increased VPS35 protein levels approximately 1.5 fold (****p < 0.0001) and 2.5 fold (****p < 0.0001), respectively, in the striatum (Fig. 1B & D), and approximately 2 fold (**p < 0.0023) and 2.3 fold (***p = 0.0009), respectively, in the SN (Fig. 1D–E). We previously demonstrated that a magnitude of increase in VPS35 protein levels of about 2-fold in the spinal cord with daily IP injections of 2a is associated with neuroprotection in an ALS mouse model [25], and thus selected 10mg/Kg for the current studies.

### The pharmacological chaperone 2a rescues motor defects in αSyn mice

**Figure 2:**
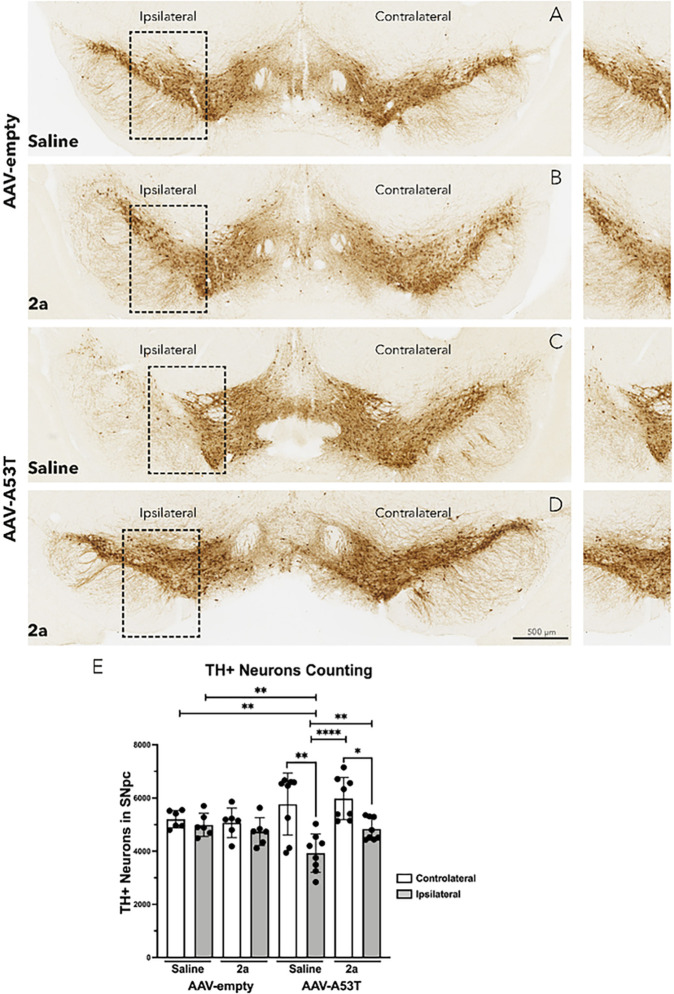
Treatment with 2a protects DA neurons in the SNpc of AAV-A53T-αSyn mice: A-D) TH immunohistochemistry of representative SN sections in AAV-A53T-SNCA and AVV-empty groups male mice treated with daily IP injections of 2a (10 mg/Kg for 100 days) or saline at 120 days post-injection. Insets in the left panels are highlighted in the right panels, scale bar is 500 mm. E) Graph shows the number of TH-positive neurons in the contralateral (white) and ipsilateral (gray) SNpc (n=6–8 mice per group). Significance determined by one-way ANOVA. Error bars represent mean ± SD.

We generated the PD mouse model by injecting AAV-A53T-αSyn or an AAV-empty vector control unilaterally in the SN and then 20 days later we began daily IP administration of 2a (or Saline solution as control) for 100 days (Fig. 2F). After 93 days of IP injections (113 days post viral vector injection) we assessed motor function on the rotarod test (Fig. 1G). We found a significant decrease (****p < 0.0001, Fig. 1G) in the latency to fall for AAV-A53T-αSyn treated daily with saline, whereas this deficit was prevented by daily treatment with 2a (**p = 0.002, indicating that 2a treatment significantly rescued the αSyn-induced motor deficit.

One week after the rotarod test (after 100 days of daily 2a IP injections) αSyn-induced asymmetry of forelimb use was assessed by the cylinder test (*30*). The AAV-A53T-αSyn injected mice treated with saline showed a significant reduction (****p < 0.0001, Fig. 1H) in use of the contralateral forelimb compared to control mice injected with AAV-empty vector and subsequently treated with saline. The deficit in contralateral forelimb use in the AAV-A53T-αSyn injected mice was significantly rescued by administration of 2a (****p < 0.0001, Fig. 1H). Two additional video files recorded during the execution of the cylinder test analyzing the asymmetry in AAV-A53T-αSyn injected mice treated with saline compared to AAV-A53T-αSyn injected mice treated with 2a compound, show these in more details (see Additional file 1 and Additional file 2). In the video we counted the number of touches of the inner surface of the cylinder with either the right (ipsilateral), the left (contralateral) or both forelimbs simultaneous. AAV-A53T-αSyn injected mice treated with saline were lethargic, with a tremor and predominantly they were using the ipsilateral forelimb (see Additional file 1). Conversely, AAV-A53T-αSyn injected mice treated with 2a 10 mg/Kg for 100 days were more active when added to the cylinder and the spontaneous forelimb use was more intense and simultaneous indicating a rescue in the asymmetry associated to this PD mouse model (see Additional file 2).

### 2a protects DA neurons in the SNpc of αSyn mice

We next assessed the effect of 2a on DA neuronal loss in the SNpc (Fig. 2A–E) by quantifying tyrosine hydroxylase immunoreactive (TH+) neurons in the SNpc (Fig. 2). In the control group (AAV-empty vector mice treated with saline) there was no significant loss of TH + neurons on the injected side compared to the contralateral side (Fig. 2A & E). Administration of 2a at 10 mg/Kg for 100 days in the control group did not change the number of TH + neurons (Fig. 2B & E). 120 days after unilateral AAV-A53T-αSyn injection, saline-treated mice showed a significant ~ 35% reduction of TH + neurons ipsilateral to the AAV-injected side compared to the contralateral side (**p = 0.019, Fig. 2C & E), as well as a significant decrease compared to TH + neurons of both the ipsilateral (**p = 0.0018, Fig. 2A & E) and contralateral sides (**p = 0.0078, Fig. 2A & E) of the AAV-empty vector saline treated controls. Administration of 2a for 100 days in AAV-A53T-αSyn injected mice significantly attenuates the loss of DA (TH+) neurons (*p < 0.0029, Fig. 2D & E).

### 2a protects striatal DA fibers loss in αSyn mice

**Figure 3:**
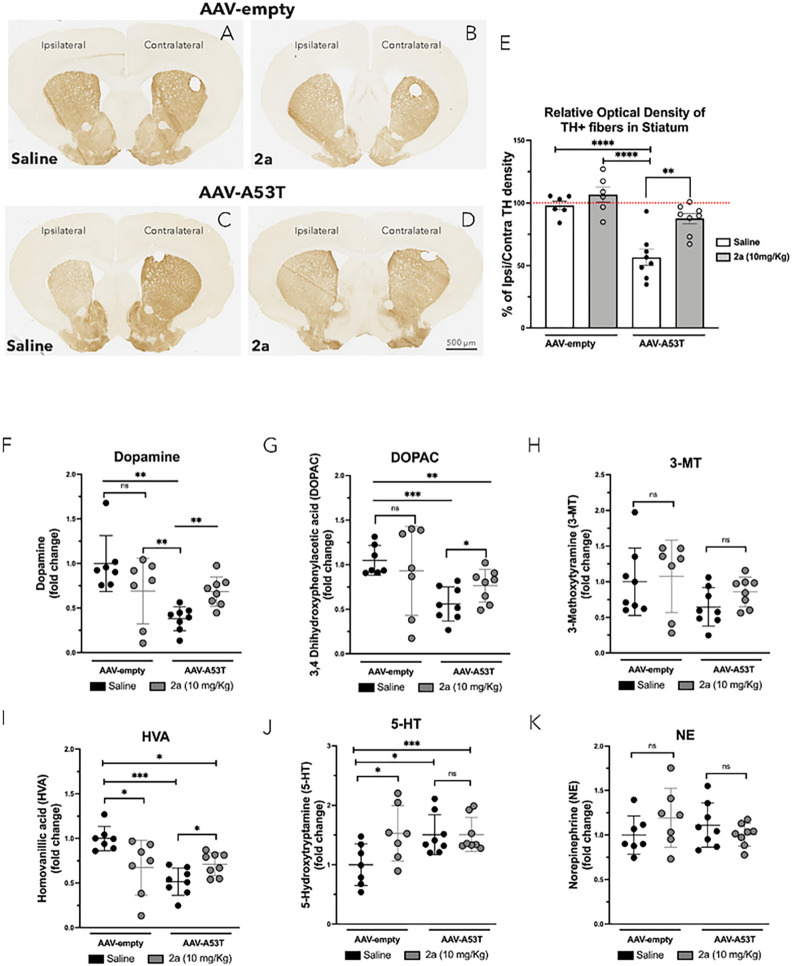
Treatment with 2a protects against loss of striatal DA fibers and monoamines in AAV-A53T-αSyn injected mice: A-D) TH immunohistochemistry in representative striatal sections of male mice treated with 2a (10 mg/Kg for 100 days) at 120 days post-injection of AAV-A53T-SNCA, scale bar is 500 mm. E) Relative optical density of TH+ fibers in the ipsilateral striatum compared with the contralateral side of male mice in each group (n=6–8 mice per group). Significance determined by one-way ANOVA. Error bars represent mean ± SD F-K) Evaluation of ipsilateral striatal monoamine in mice treated with 2a (10 mg/Kg for 100 days) or saline solution at 120 days post-injection of AAV-A53Tor AAV-empty as control; F) mean DA levels, G) mean of 3,4-Dihydroxyphenylacetic acid levels (DOPAC), H) mean levels of 3-methoxytyramine (3-MT), I) mean levels of homovanillic acid (HVA), J) mean levels of 5-hydroxytryptamine (5-HT) and K) mean levels of Norepinephrine (NE); the values are expressed as fold change (7–9 mice per group). Significance determined by one-way ANOVA. Error bars represent mean ± SD.

To test the impact of 2a on αSyn-induced loss of DA neurites projecting to the striatum, we measured the density of TH + terminals in the striatum of brain sections (Fig. 3A–E). Notably, the relative immunostaining intensity of TH + fibers was significantly decreased by nearly 50% in AAV-A53T-αSyn mice treated with saline (****p < 0.0001, Fig. 3C & E) compared to control AAV-empty vector injected mice treated with saline (Fig. 3A & E) and compared to AAV-empty vector mice treated with 2a (Fig. 3B & E). Daily IP administration of 2a (10 mg/Kg) for 100 days in AAV-A53T-αSyn mice significantly protected against loss of striatal TH + fibers (**p < 0.001, Fig. 3D & E). These results show that 2a protects against αSyn-induced loss of DA neurons in the SNpc and against loss of TH + striatal terminals in a mouse model of PD.

### 2a protects against loss of striatal DA and DA metabolites in αSyn mice

We analyzed levels of DA and its metabolites including 3,4-dihydroxyphenylacetic acid (DOPAC), 3-methoxytyramine (3-MT) and homovanillic acid (HVA) in the ipsilateral striatal hemisphere of AAV-A53T-αSyn mice compared to the control AAV-empty vector mice treated with saline or 2a (Fig. 3F–I). Levels of DA were significantly reduced by about 60% in AAV-A53T-αSyn mice treated with saline compared AAV-empty mice treated with saline (**p < 0.0078, Fig. 3F) and by about 50% compared to AAV-empty mice treated with 2a (**p < 0.004, Fig. 3F). Treatment with 2a protected AAV-A53T-αSyn mice against this αSyn-induced loss of striatal DA (**p < 0.0068, Fig. 3F). Furthermore, 2a administration prevented the decline of DA metabolites DOPAC (*p = 0.034, Fig. 3G) and HVA (*p = 0.0147, Fig. 3I) with only a non-significant trend for 3-MT (Fig. 3H). We found a significant increase in striatal serotonin (5-HT) upon administration of 2a (compared to saline) in AAV-empty mice, but no effect of 2a versus saline on 5-HT levels in the AAV-A53T-αSyn injected mice (Fig. 3J). No effects from AAV-A53T-αSyn or from 2a treatment were seen for striatal norepinephrine (NE, Fig. 3K). These results show that the AAV-A53T-αSyn PD mouse model recapitulates key features of PD, including motor behavioral deficits, degeneration of DA neurons in the SNpc, loss of striatal TH + fibers, and reduction in the striatal DA and DA metabolites. Furthermore, these results demonstrate significant protection from 2a treatment against each of these Syn induced deficits.

### 2a increases retromer protein levels and function in the SNpc of αSyn mice

**Figure 4:**
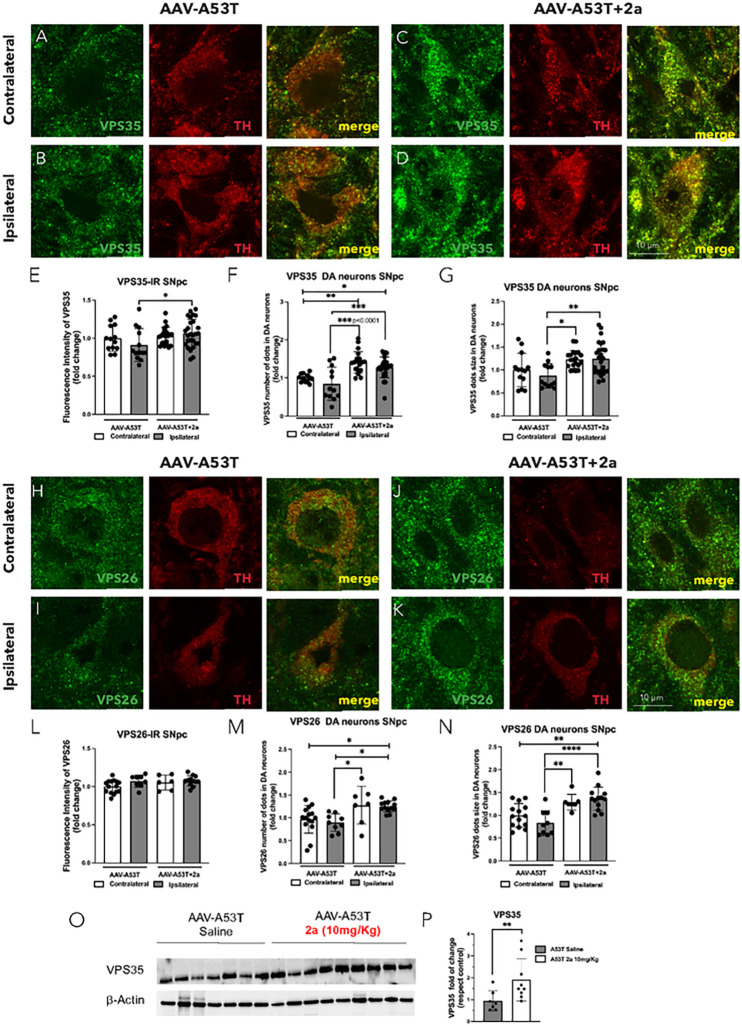
The pharmacological chaperone 2a increases retromer subunit protein immunoreactivity (IR), number of puncta and puncta size in DA neurons of the SN in AAV-A53T mice: A-D) Double immunofluorescence of VPS35 (green) in TH-positive neurons (red) in the contralateral and ipsilateral SNpc of AAV-A53T injected mice treated with saline (A-B), or 2a (C-D), magnification 240X and scale bar is 10 mm; E-G) relative quantification of VPS35 fluorescence intensity (fold change) (E), number of VPS35 puncta where each dot represents an individual DA neuron in the SNpc (F) and VPS35 puncta size (G); H-K) Double immunofluorescence of VPS26 (green) in TH-positive neurons (red) in the contralateral and ipsilateral SNpc of AAV-A53T injected mice treated with saline (H-I), or 2a (J-K), magnification 240X and scale bar is 10 mm; L-N) relative quantification of VPS26 fluorescence intensity (fold change) (L), number of VPS26 puncta (M) and VPS26 puncta size (N); for these analyses we used 4–8 mice 3 fields each. O-P) Immunoblot analysis of VPS35 fold change in ipsilateral SN of AAV-A53T mice daily IP injected with 2a (10 mg/Kg) or saline for 100 days normalized to b-Actin protein levels and relative densitometric analysis; for this analysis (n=7–9 mice per group). Significance determined by one-way ANOVA. Error bars represent mean ± SD.

**Figure 5:**
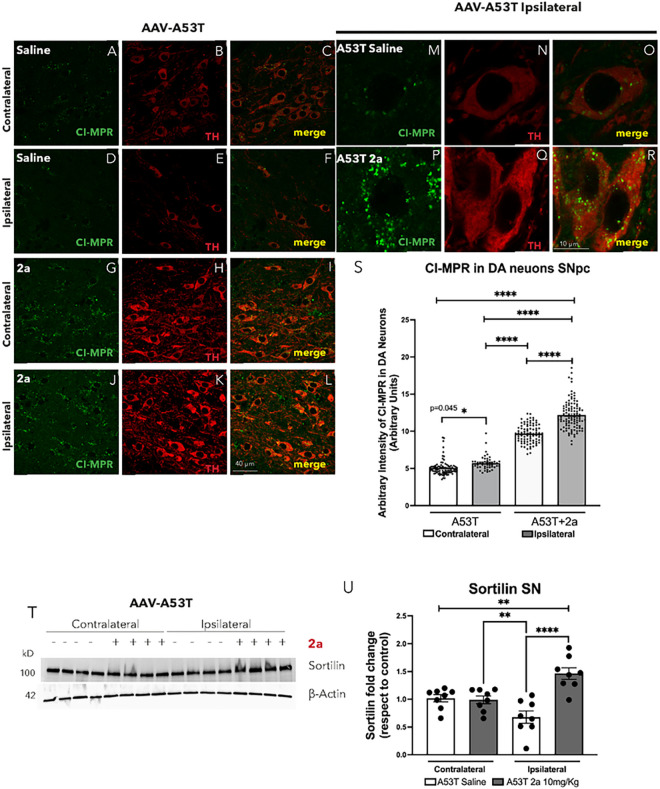
The pharmacological chaperone 2a increases SN retromer cargo protein levels in the SN of AAV-A53T injected mice: A-L) Double immunofluorescence of CI-MPR (green) in TH-positive neurons (red) in the SNpc in contralateral and ipsilateral AAV-A53T injected mice treated with saline (A-D), or 2a (G-L) magnification 120X and scale bar is 40 mm; M-S) double immunofluorescence of CI-MPR (green) in TH-positive DA neurons (red) in the ipsilateral SNpc of AAV-A53T injected mice treated with saline (M-O) or 2a (P-R) at higher magnification 240X and scale bar is 10 mm; S) Graph indicates the fluorescence intensity (arbitrary units) of CI-MPR in DA neurons in SNpc, each dot represents an individual DA neuron in the SNpc (n=6–8 mice per group); T) Western blot of Sortilin protein levels in contralateral and ipsilateral SN brain extracts from AAV-A53T injected mice; U) relative Sortilin protein levels normalized to b-Actin (8 mice per group) in the ipsilateral and contralateral SN of AAV-A53T injected mice treated with saline (white) or 2a (grey) (n=4 mice per group); Significance determined by one-way ANOVA. Error bars represent mean ± SD.

We assessed the impact of 2a on VPS35 and VPS26 immunoreactivity in DA neurons in SNpc in AAV-A53T-αSyn mice by immunofluorescence (IR) (Fig. 4). We found a significant increase in VPS35-IR (Fig. 4, A–D & E, *p = 0.0494), number of VPS35 puncta (Fig. 4A–D & F, ***p = 0.0004) and VPS35 puncta size (Fig. 4A–D & G, ****p < 0.0001) in ipsilateral DA neurons of A53T mice treated with 2a (10mg/Kg for 100 days) compared to ipsilateral A53T mice treated with saline. 2a led to a significant increase in the number of VPS26 puncta (H-K & M, *p = 0.00174) and VPS26 puncta size (Fig. 4, H–K & N, ****p < 0.0001) in ipsilateral DA neurons of A53T mice treated with 2a compared to ipsilateral A53T mice treated with saline. We also assessed if the significant increase in number of VPS35/VPS26 puncta and VPS35/VPS26 puncta size is associated with a boost in retromer function by analyzing two well-know VPS35 cargoes, Sortilin and CI-MPR (Fig. 5) [28–29]. These 2 cargo proteins previously have been found to be increased in MNs of SOD1G93A mice by treatment with 2a [25]. We analyzed CI-MPR-IR in the SNpc of AAV-A53T-αSyn mice treated with 2a or saline solution as control in the contralateral and ipsilateral hemispheres (Fig. 5A–L), and specifically in DA neurons ipsilateral to the AAV injection (Fig. 5M–R). We found a slight but statistically significant increase in CI-MPR immunoreactive intensity in TH + neurons in the ipsilateral SNpc of AAV-A53T-αSyn injected mice (p = 0.045), with a larger magnitude increase induced by treatment with 2a (p < 0.0001, Fig. 5M–S). Sortilin protein levels in the SN were analyzed by immunoblot and we found a significant ipsilateral reduction in Sortilin immunoreative intensity in AAV-A53T-αSyn injected mice treated with saline compared to the contralateral (non-injected) side (**p < 0.0078, Fig. 5T–U). Administration of 2a in AAV-A53T-αSyn injected mice completely prevented this αSyn-induced reduction in Sortilin (****p < 0.0001, Fig. 5T–U). Together, these data suggest that 2a treatment increases retromer subunit levels as well as retromer function in the SN.

### 2a attenuates the accumulation of pathological αSyn in the SNpc of αSyn mice

**Figure 6:**
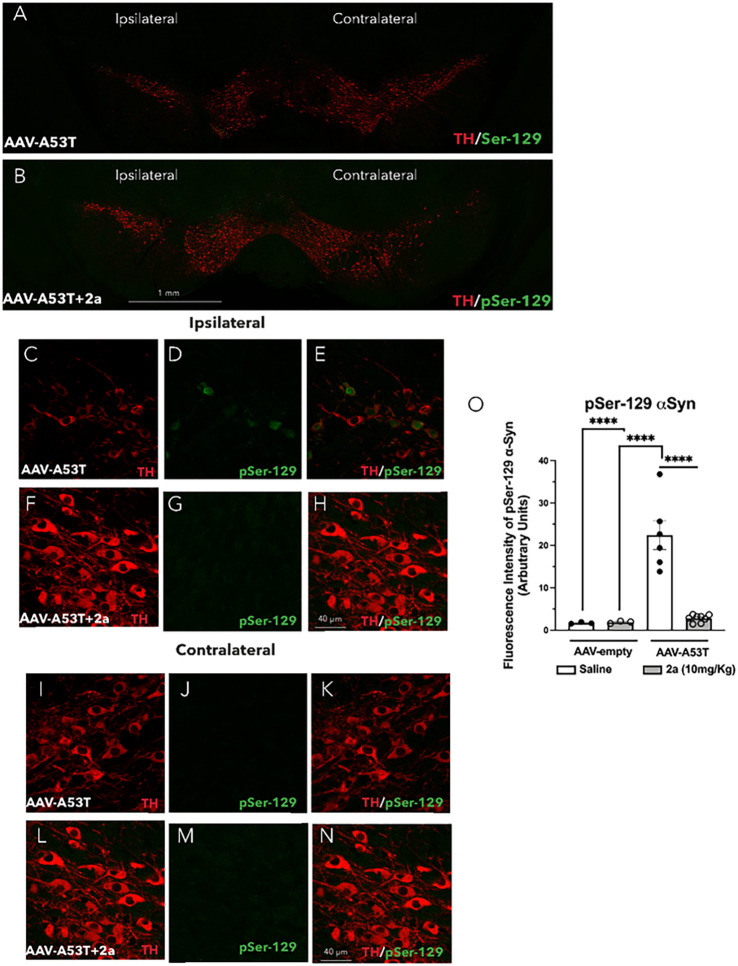
Treatment with 2a protects DA neurons and rescues αSyn accumulation in the SNpc of AAV-A53T-αSyn injected mice: A-N) Immunofluorescence in representative SN sections following double labeling of TH-positive neurons (red) and phospho-Ser129 αSyn (pSer-129, green) in AAV-A53T-αSyn injected mice (n=7–8 mice per group) treated with daily IP injections of saline or 2a at 10 mg/Kg for 100 days; A-B) whole SN section of AV-A53T-αSyn injected mice treated with saline (A) or 2a (B), scale bar is 1 mm; C-H) double immunolabeling of TH-positive neurons and pSer-129 ipsilateral SN of AV-A53T-αSyn injected mice treated with saline (C-E) or 2a (F-H), magnification 120X and scale bar is 40 mm. O). Quantification of average pSer-129 αSyn fluorescence intensity in ipsilateral DA neurons in the SNpc of AAV-empty or AAV-A53T-αSyn injected mice treated with saline (white) or 2a (gray). Significance determined by ANOVA. Error bars represent mean ± SD.

Prior studies have shown that a genetic manipulation to increase VPS35 protein levels leads to a reduction in the accumulation of αSyn aggregates in the hippocampus of a PD mouse model overexpressing human wild type αSyn [19]. Here we analyzed the effect of pharmacological stabilization of the retromer complex by treatment with 2a by daily IP injections for 100 days in AAV-A53T-αSyn mice (Fig. 1F). We analyzed αSyn immunoreactivity in DA neurons of the SNpc, labeling TH + neurons (red) and phospho-serine-129 αSyn (pSer-129, green) by double immunofluorescence (Fig. 6A–N). We found a robust increase in the fluorescence intensity of pSer129 αSyn-IR (≈ 20 folds increase) in the ipsilateral SNpc of AAV-A53T-αSyn injected mice treated with saline (****p < 0.0001, Fig. 6A, C–D & O), indicating the accumulation of pathological αSyn in individual DA neurons. Treatment with 2a significantly attenuated pathological pSer129 αSyn accumulation in the SNpc of these mice (****p < 0.0001, Fig. 6B, F–H & O). We did not detect pSer129 αSyn immunoreactivity in the contralateral SNpc of AAV-A53T-αSyn injected mice treated with saline (Fig. 6A & I–K) or with 2a (Fig. 6B & L–N).

**Figure 7:**
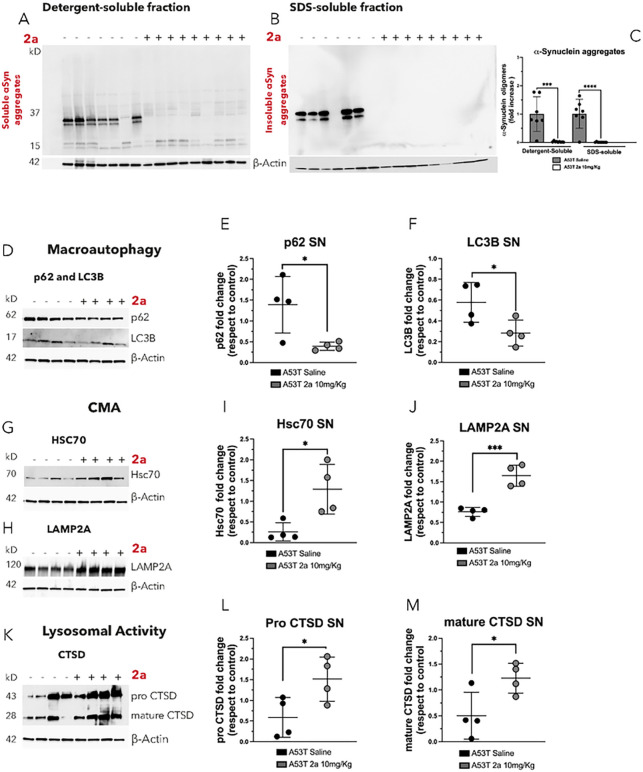
Treatment with 2a reduces αSyn aggregates and boosts αSyn degradation pathways in the SN of AAV-A53T-αSyn injected mice: A-B) Evaluation of 2a efficacy in reducing soluble αSyn aggregates in the detergent-soluble fraction (A) and insoluble αSyn aggregates in the SDS-soluble fraction in the SN of AAV-A53T-αSyn injected mice (n=7–9 mice per group) by western blot. C) Densitometric analysis of αSyn aggregates in the SN of AAV-A53T-αSyn injected mice normalized to b-Actin protein levels. D-M) 2a effect on αSyn degradation pathways in ipsilateral SN of AAV-A53T-αSyn injected mice (n=4 mice per group) by immunoblot analysis normalized to b-Actin protein levels; D-F) 2a effect on macroautophagic markers (p62 and LC3B) protein levels (D) and relative densitometric analysis of p62 (E) and LC3B (F); G-J) 2a effect on CMA markers (Hsc70 and LAMP2A) protein levels and relative densitometric analysis of Hsc70 (I) and LAMP2A (J); K-M) 2a effect on CTSD (pro and mature forms) and relative densitometric analysis of pro CTSD (L) and mature CTSD (M). Significance determined by ANOVA. Error bars represent mean ± SD.

We also analyzed the effect of 2a versus saline on the clearance of αSyn aggregates in the ipsilateral SN of AAV-A53T-αSyn injected mice by immunoblot (Fig. 7A–C). Brain extracts from the ipsilateral SN of AAV- 53T-αSyn mice were dissected, fractionated by ultracentrifugation (100K×g for 45 minutes) into detergent-soluble and SDS-soluble fractions and αSyn aggregates were analyzed and normalized to β-actin protein levels (Fig. 7A–C). The SN of AAV-A53T-αSyn mice had substantial amounts of soluble αSyn oligomers (Fig. 7A) and insoluble αSyn aggregates extracted using SDS detergent (Fig. 7B). Administration of 2a resulted in a strong clearance of αSyn aggregates in both the detergent-soluble (***p < 0.0001, Fig. 5A & D) and the SDS-soluble fractions (***p < 0.0001, Fig. 5B & D), confirming the results of the double-immunolabeling (Fig. 6). Together, these results demonstrate a strong effect of 2a treatment in attenuating the accumulation of αSyn in the SN.

### 2a boosts αSyn degradation pathways in the SN of αSyn mice

We hypothesized that the reduction of αSyn induced by 2a treatment may reflect an impact of 2a on αSyn degradation pathways. It has been already reported that VPS35 interact with WASH-FAM21 subunit to control of autophagosome formation in the late stage of the macroautophagic process [31], and VPS35 it is a key component of the CMA activity by controlling the retrograde trafficking of LAMP2A receptor [20]. Therefore, we analyzed the effect of the pharmacological chaperone 2a on αSyn degradation pathways, macroautophagy, chaperone mediated autophagy (CMA) and the endo-lysosomal pathway in the SN of AAV-A53T-αSyn injected mice treated with saline or 2a by immunoblot analysis (Fig. 7D–M). p62 and LC3B are two macroautophagic markers and their protein levels increased in autophagy impairments as well as well as LC3-positive and p62-positive structures have been found prominent in autophagy-deficient cells [32]. LC3B and p62 both were significantly decreased in ipsilateral SN brain extracts of AAV-A53T-αSyn injected mice treated with 2a compared to control mice treated with saline (*p = 0.026 for p62, Fig. 7D & E, and p = 0.041 for LC3B, Fig. 7D & F). Treatment with 2a in AAV-A53T-αSyn mice resulted in significant increases in CMA markers (Hsc70 and LAMP2A) compared to saline controls (*p = 0.017 for Hsc70, Fig. 7G & I; **p = 0.0013 for LAMP2A, Fig. 7H & J). We also assessed the effect of 2a on lysosomal activity by analyzing Cathepsin D (CTSD) protein levels, the main lysosomal hydrolase involved in αSyn degradation. We found that 2a treatment, compared to saline-treated controls, increased both pro-CTSD (p = 0.028, Fig. 7K–L) and mature CTSD (p = 0.0447, Fig. 7K & M) in the ipsilateral SN of AAV-A53T-αSyn mice.

These data indicate that 2a treatment leads to activation of macroautophagy, an increase in CMA, and to increases in lysosomal functions, which are pathways that are known to regulate αSyn degradation [10]. These data suggest that 2a may lead to neuroprotection by promoting clearance of αSyn through activation of these degradation pathways, although further studies will be needed to confirm this hypothesis.

## Discussion

In the present study we assessed the neuroprotective effects of a pharmacological approach to stabilize the retromer complex [25] in a PD mouse model overexpressing mutant (A53T) αSyn in the SN [30]. We found that 2a treatment increases VPS35 immunoreactivity (Fig. 4), the number of VPS35/VPS26 puncta and puncta size (Fig. 4) and retromer functions (Fig. 5) in DA neurons in the SNpc of AAV-A53T-αSyn mice. This pharmacological approach rescues behavioral deficits (Fig. 1G–H), protects against loss of DA neurons in the SNpc (Fig. 2), and attenuates loss of striatal DA fibers (Fig. 3A–E) and striatal monoamines (Fig. 3F–H) in AAV-A53T-αSyn mice. Notably, the increase in functions of the retromer complex was associated with a robust reduction of pathological pSer129-αSyn in DA neurons of the SNpc (Fig. 6), as well as rescues in soluble and insoluble total αSyn accumulation (Fig. 7) in the SN.

The molecular mechanisms leading to neuroprotection by VPS35 against αSyn toxicity are still unclear but recent studies highlight a pivotal role for VPS35 in control of the main αSyn degradation pathways [10]. First, VPS35 is a key modulator of autophagosome formation in the late stage of the macroautophagic process [31] and we now report an increase in macroautophagy upon treatment with

2a in AAV-A53T-αSyn mice, characterized by a significant reduction in the autophagic markers, p62 and LC3B, in the ipsilateral SN (Fig. 7D–F). Furthermore, VPS35 mediates retrieval transport of the LAMP2A receptor, a key protein for CMA, to lysosomes [20]; therefore, the 2a-induced significant increase in CMA markers (LAMP2A and Hsc70) in the ipsilateral SN of AAV-A53T-αSyn mice (Fig. 7G–J) indicates that 2a also activates CMA. Finally, VPS35 acts on regulating lysosomal homeostasis [33] and lysosomal-degradation system by managing the sorting of lysosomal hydrolase receptors [29], as well as Sortilin and CI-MPR, that we found to be significantly increased in the ipsilateral SN by treatment with 2a in AAV-A53T-αSyn mice (Fig. 5). These lysosomal hydrolase receptors act directly in the sorting of misfolded proteins [29] and can regulate the clearance of misfolded proteins indirectly by controlling trafficking and maturation of lysosomal hydrolases that lead to misfolded protein degradation [29]. Sortilin and CI-MPR, control trafficking and maturation of CTSD, the major enzyme that leads to the degradation of αSyn [34], from the trans-Golgi network to lysosomes. We found that administration of 2a significantly increased pro and mature forms of CTSD protein levels in the ipsilateral SN of AAV-A53T-αSyn mice (Fig. 7K–M). Together these data showed that the 2a-induced increase in retromer protein levels and functions results in a boost of lysosomal activity and promotes the clearance of pathological and aggregated forms of αSyn.

Recently retromer dysfunction has been linked to different neurodegenerative disorders, including ALS [25] and AD [23]. A significant reduction in levels of retromer subunits (VPS35 and VPS26) has been reported in IPSC-derived motor neurons (MNs) from ALS patients, in the ventral horn MNs from ALS post-mortem explants and in MNs from an ALS mouse model (SOD1^G93A^) [25]. Furthermore, neuron-specific deletion of VPS35 leads to the selective degeneration of ventral horn MNs in the spinal cord accompanied by protein inclusions and marked gliosis [35]. We previously showed that pharmacological stabilization of the retromer complex by treatment with 2a in an ALS mouse model (SOD1^G93A^ mice) increases retromer subunit protein levels (VPS35, VPS29 and VPS26) and markers of retromer functions (Sortilin and CI-MPR) and protects against degeneration of spinal cord ventral horn MNs [25]. We now demonstrate that retromer stabilization by 2a in the AAV-A53T-αSyn PD mouse model promotes the clearance of pathological misfolded aggregates of αSyn, (Fig. 6 and Fig. 7) and protects DA neurons from degeneration. In support of a crucial role of VPS35-retromer subunit in controlling protein aggregation and accumulation of misfolded proteins, previous studies have been shown that depletion of VPS35 leads to misfolded protein accumulation in *yeast* [19].

Chen and collaborators have been shown that genetic manipulations of VPS35, did not rescue αSyn-induced neurotoxicity and VP35 and αSyn fail to interact and modulate neurodegeneration [36]. In contrast, a prior study showed that using lentiviruses to increase VPS35 induces neuroprotection and a reduction in αSyn accumulation in the hippocampus of αSyn overexpressing mice [19]. Furthermore, two independent studies on induced pluripotent stem cell-derived neurons patients harboring the p.D620N mutation have shown αSyn pathology and clearance impairment [19, 37]. VPS35 deficient mice resulted in the accumulation of αSyn aggregates [14], and recently a VPS35 knock-in mice recapitulate a spectrum of cardinal features of PD including a dramatically accumulation of αSyn in SNpc from 15 month of age without Tau pathology [38]. A case report of atypical parkinsonism associated with rare sequence variants in VPS35 (c.102 + 33G > A) and FBX07 showed Lewy bodies in SN and Syn immunoreactivity in midbrain, pons, medulla, basal ganglia, amygdala, hippocampus and neocortical cortex [39]. The current study shows that a pharmacological stabilizer of VSP35 leads to potent neuroprotection with robust attenuation of pathological αSyn accumulation in the SN in a mouse model of PD, thus supporting the potential testing of pharmacological strategies to target VSP35 for neuroprotection in PD. However, although we have demonstrated that compound 2a increases VPS35 and retromer functions, further studies are needed to assess the possibility that 2a also could have an impact on other pathways that contribute to its neuroprotective effects.

No strategies have yet been proven clinically to slow or block the progression of PD. Mutations in the VPS35 gene are a rare autosomal dominant cause of familial PD [7 – 6] and accordingly, VPS35 controls mechanisms of relevance to PD pathogenesis: i) pathways for clearance of abnormal proteins, including αSyn aggregates [19–21], ii) mitochondrial function by regulating mitochondrial dynamics [16–18], and regulation and maintenance of neuronal signaling events [11–13]. The data presented here in a pathophysiologically relevant αSyn-based PD mouse model highlight the neuroprotective potential of increasing retromer levels to block the accumulation of pathological αSyn and to protect against neurodegeneration in PD.

However, an in-depth analysis is needed to precisely elucidate the molecular mechanism/s controlled by the retromer stabilization that leads to the protection of DA neurons and the clearance of toxic αSyn aggregates. Transcriptomic and proteomic studies may be helpful to evaluate the potential effects of 2a on other pathways that may act in parallel or synergistically with retromer stabilization to contribute to the neuroprotective effect.

## Conclusion

Treatment with 2a, a VPS35-targeting small molecule (pharmacological chaperone), leads to robust protection against αSyn toxicity in an AAV-A53T- αSyn model of PD. These data support the potential for future clinical testing of 2a or other VPS35-targeted therapies for disease-modifying effects in PD.

## Data Availability

All key data are available in the main test. Any additional raw data from these studies will be provided upon request.

## Abbreviations

αSyn: α-synuclein
2a: 1,3 phenyl bis guanylhydrazone
VPS35: vacuolar sorting protein 35
VPS26: vacuolar sorting protein 26
CMA: chaperone-mediated autophagy
CTSD: Cathepsin D
DA: dopaminergic
IP: intra peritoneal
PD: Parkinson’s disease
SNpc: substantia nigra pars compacta
ST: Striatum
TH: Tyrosine Hydroxylase
